# Advancing annually resolved oxygen isotope dendrochronology to overcome wood-dating limitations in the Euro-Atlantic region

**DOI:** 10.1371/journal.pone.0357031

**Published:** 2026-09-23

**Authors:** Clara Rodríguez-Morata, Darren Davies, Neil J. Loader, Danny McCarroll, Sjoerd Van Daalen, Kristof Haneca, Valerie Daux, Marta Domínguez-Delmás

**Affiliations:** 1 Naturalis Biodiversity Center, Leiden, The Netherlands; 2 School of Biosciences, Geography and Physics, Swansea University, Swansea, United Kingdom; 3 Van Daalen Dendrochronologie, Wijhe, The Netherlands; 4 Flanders Heritage Agency, Brussels, Belgium; 5 Laboratoire des Sciences du Climat et de l’Environnement, CEA, CNRS, UVSQ, Université Paris-Saclay, Gif-sur-Yvette, France; 6 Cultural Heritage Agency of The Netherlands, Amersfoort, The Netherlands; Technical University in Zvolen, SLOVAKIA

## Abstract

Stable oxygen isotope dendrochronology has emerged as a ground-breaking technique for dating historic timbers when conventional tree-ring-width dendrochronology falls short. In the Euro-Atlantic region, thousands of timbers in historic buildings and archaeological sites from the Late Medieval and Early modern periods contain few tree rings or exhibit complacent growth (i.e., minimal year-to-year variation), making them undatable with conventional ring-width dendrochronology. Here, we present a 300-year-long annually-resolved δ^18^O reference chronology for the Eastern Netherlands-Northwest Germany region (TWR δ^18^O), covering the period 1300–1600 CE. We explore its correlations with other European δ^18^O chronologies, and its potential for dating short and complacent tree-ring series. We found that the TWR δ^18^O chronology exhibits strong coherence with independent δ^18^O records from Belgium and the UK, and weaker correlations with French and more continental chronologies. Our results demonstrate that δ^18^O dendrochronology can successfully date single timbers with as few as 30 rings when a local reference δ^18^O chronology is available. In the absence of such data, combining δ^18^O series from 3–5 coeval samples and extending the temporal coverage improves dating success through correlations with regional chronologies. These findings provide guidance for sampling strategies and the development of δ^18^O chronologies in the Euro-Atlantic region. The dating of currently undatable samples and their integration into dendrochronological repositories will enable a more accurate reconstruction and interpretation of past construction activities, timber trade, and forest management practices, ultimately transforming our understanding of post-medieval timber use and environmental history.

## Introduction

Dendrochronology is commonly used to provide absolute dates for historical and archaeological timbers, determine the geographical provenance of wood, and investigate environmental and climatic change through time [1–7]. However, conventional dendrochronology relies on several conditions to be successful. A relatively large number of annual rings and a growth pattern strongly controlled by external forcing (i.e., environmental conditions) generally increase the likelihood of successful statistical cross-dating [8]. As a result, tree-ring reference datasets and sampling strategies are often biased towards timbers containing relatively long ring sequences, as these generally provide greater statistical confidence for dating [9–11]. Samples with few tree rings are usually left unsampled, and when sampled, often remain undated, restricting their incorporation into dendrochronological datasets. The simultaneous development of stable oxygen isotope (δ^18^O) dendrochronology in the UK and Japan addresses many of these challenges [12–15], and has demonstrated the suitability of this technique in Europe and Southeast Asia for dating historic timbers in situations when conventional (tree-ring width) dendrochronology is unsuccessful or cannot be applied [16–21]. Timbers derived from such young (less than 70 years) and fast-growing trees make up to 70% of timbers in historic buildings dating from the 13th to the 17th centuries in the Euro-Atlantic region [22–25], and up to 85% in Atlantic-Iberian shipwrecks [26]. Such timbers are usually left unsampled or unstudied, restricting our knowledge about the material culture and our understanding of wood supply for construction in the Euro-Atlantic region. Therefore, the development of reference chronologies of δ^18^O for this region has become a pressing endeavour, as it would enable the exact dating of the currently unexplored and massive volume of undated historical timbers from historic buildings and archaeological sites, while also offering the potential for interspecific dating.

Here, we present a 300-year δ^18^O chronology built with samples from the Eastern Netherlands or north western Germany (Twente-Westfalia region; hereafter TWR δ^18^O chronology) found in roof structures of historic buildings in The Netherlands. The historical region of Twente-Westphalia supplied timber for artworks, furniture, shipbuilding and other construction purposes for much of The Netherlands and north western Germany since at least the Middle Ages [5,27,28]. We explore the geographic spread of the climate (dating) signal of the TWR δ^18^O chronology by comparing it with adjacent δ^18^O chronologies, and test its dating potential using δ^18^O series from timbers of the Jerome’s Old Church in Noordwijk, The Netherlands [29]. The Noordwijk church timbers (NWK) provide an interesting case study for testing stable oxygen isotope dendrochronology dating, as they contain few tree rings (30–52), show complacent growth, and have already been dated. In study [29], two timber samples from Noordwijk Jerome’s Old Church, previously dated using conventional ring-width dendrochronology, were used to construct an annually-resolved site record covering the period 1355–1435 CE. The authors developed δ^18^O series from three undated timbers with 52, 49 and 30 rings and were then securely dated against this site δ^18^O record constrained by ring width dendrochronology. This result demonstrated that δ^18^O dendrochronology has the potential to date timbers with very few tree rings when reference isotopic data is available for the source area of the wood. Testing the dating potential of our new TWR δ^18^O chronology with the Noordwijk dataset and comparing both datasets with adjacent δ^18^O chronologies will increase understanding of the geographical spread of the isotopic signal, and explore the potential of the technique to determine wood provenance thereby informing strategies for the development of this and further δ^18^O chronologies across the Euro-Atlantic region.

## Methods

### Development of the TWR δ^18^O chronology

**Fig 1:**
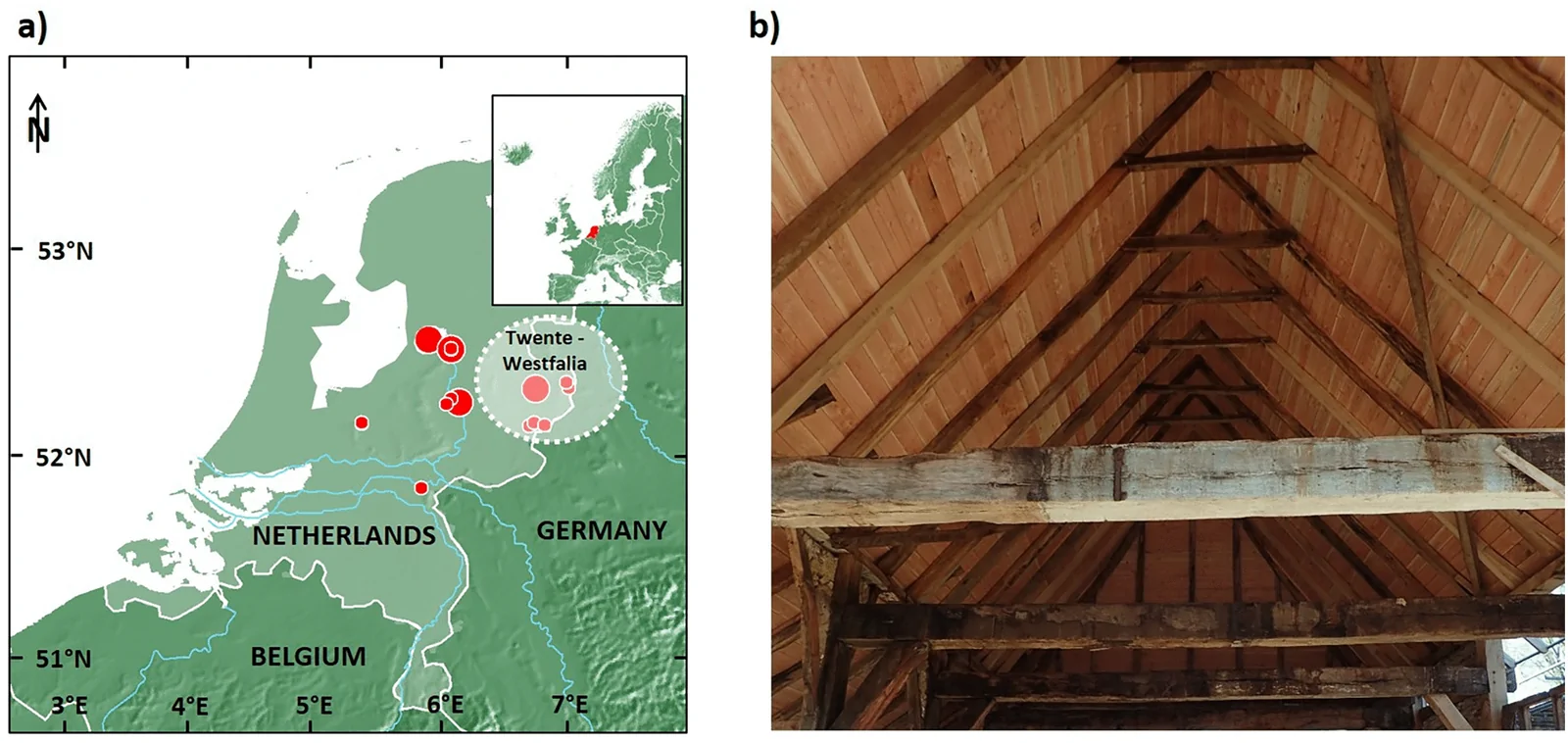
Regional context of the TWR δ^18^O chronology. a) Geographic location of the buildings from which the timber samples were collected. Bigger circles indicate several samples of different buildings in the same town. White circle indicates the likely source area of the timber. Base map created by the authors using publicly available geospatial datasets. National boundaries from GISCO (Eurostat), river network from Natural Earth, and hill shade from the European Environment Agency (EEA). Sampling locations and other thematic layers were generated by the authors. b) Roof structure of one of the historic buildings contributing to the TWR δ^18^O chronology, Hertmerweg 31, Hertme. Picture source: Sjoerd Van Daalen.

To develop the annual δ^18^O chronology for the Twente-Westfalia Region spanning 1300–1600 CE, pencil-shaped core oak (*Quercus* spp.) samples from 22 buildings across northern Netherlands (Fig 1a, b) were used. No permits were required for the described study, which complied with all relevant regulations. These samples had previously been dated and their provenance established as eastern Netherlands/north western Germany through conventional dendrochronology [30]. Some dated segments had to be excluded from the isotope chronology due to wood deterioration caused by woodworm that may induce contamination, or extremely narrow rings that prevented reliable early wood–latewood separation. Additional segments were also omitted in periods where chronology replication was already sufficient, allowing analytical resources to be prioritised towards under-replicated intervals. To prevent the composite from being dominated by timber sourced from the same trees or forest, we avoided pooling samples from the same building, as medieval construction practices often involved using timbers from the same trees and forests in a single construction phase [22]. Only the latewood portion of each annual ring was analysed to avoid carryover effects [31–33]. Each tree ring was carefully divided using a scalpel and the individual annual early wood and latewood portions separated. The latewood portion was then chopped into millimetric pieces and stored inside microcentrifuge tubes. Next, similar amounts of latewood from the same calendar year were pooled (i.e., combined) to develop the reference chronology [13,34,35]. The α-cellulose from the pooled wood samples was extracted using an acidified sodium chlorite solution (Jayme-Wise method) followed by hydrolysis of hemicelluloses using sodium hydroxide [36]. The method was modified from [36] replacing the free-standing Soxhlet thimbles with a block array [37] and homogenising the α-cellulose at the end of the chemical process prior to freeze drying [38]. Using a Flash HT elemental analyser interfaced with a Thermo Delta V plus mass spectrometer, samples were converted into CO gas through pyrolysis at 1400°C and δ^18^O ratios determined [39].The raw isotope data were measured relative to in-house standard Sigma Cellulose, (micro-crystalline α-cellulose): Sigma Aldrich UK (No. C-8002 Lot. 92F-0243) and the IAEA C3 holocellulose: International Atomic Energy Agency, Vienna, Austria. Sheet No. 372. This IAEA reference material is not characterized for oxygen isotopes and consequently used here to provide an independent precision and linearity check based upon the value obtained by [40] in their intercomparison. Raw data were checked for drift through the analysis of the cellulose standard materials spaced periodically throughout each run (typically 5 standards per 20 samples). No significant drift in standard values was detected and no corrections were made. Sample size was constrained to 0.30–0.35 mg of dry alpha cellulose, meaning that there was no need to make a sample size correction. Samples were run in duplicate where material permitted as per [13]. The data were indexed prior to isotope dendrochronology by subtraction after filtering of the isotope data using a 9-year rectangular filter. For the purpose of this study, analytical precision is determined based upon the replicate analysis of standards in each sample run using the standard deviation of the population. Dating and chronology building using the resulting stable oxygen isotope data followed the statistical framework [13] implemented in the ISODATE software [41].

### Regional shared signal

To assess the degree of common variability shared between the TWR δ^18^O chronology and other available European δ^18^O records, and to evaluate whether these relationships remained consistent through time (hereafter referred to as temporal stability), we computed Pearson’s correlation coefficients against five independent annually resolved δ^18^O chronologies from across Europe that differ in length and annual sample replication: Central England [13] (UK, sample replication of 10, 1200–2000 CE); Bruges [18], Belgium (BRG, sample depth between 1 and 4, 1325–1468 CE); Fontainebleau (FON, sample replication between 2 and 9, 1326–2000 CE) and Angoulême (ANG, sample replication between 4 and 6, 1360–2004 CE), France [42]; and Czech Republic – Bavaria [43] (CHBV, sample replication between 5 and 21, 75 BCE –2018 CE). Prior to correlation analysis, all chronologies were pre-whitened using a rectangular 9-year filter [13] to reduce temporal autocorrelation. Two correlation analyses were performed: (i) over the full overlapping period between the TWR chronology and each reference chronology (hereafter, correlation_1, and (ii) over the common interval shared by all chronologies (1360–1468 CE, constrained by ANG; hereafter, correlation_2). In addition, 50-year running correlations with a one-year step were calculated to assess the temporal stability of the shared signal and to evaluate the influence of changes in sample replication through time. This analysis helps identify periods during which the chronology exhibits stronger or weaker coherence with other European δ^18^O records.

### Assessment of the dating potential

To assess the potential of the TWR δ^18^O chronology to date individual and coeval timbers with few tree rings across the study region, we used δ^18^O series from roof timbers of Jerome’s church in Noordwijk, The Netherlands. These timbers have been previously dated through the development of a site-specific isotope chronology with samples dated by conventional dendrochronology [29]. Here, we test whether the same material could have been independently dated using our new TWR δ^18^O chronology and/or with the adjacent chronologies described in 2.2. For this, we used the isotope dendrochronology web application ISODATE [41]. After applying a 9-year filter, Pearson’s correlation coefficients were calculated between series and then converted into Student’s *t*-values. ISODATE identifies the most probable alignment between a test and reference chronology, taking into account the filter length, chronology autocorrelation and multiple testing (see [12] for further discussion). The probability of the chance occurrence of an r‐value is reported as the inverse of the p-value (1/*p*) capped at one million. An Isolation Factor (IF; i.e., the ratio of probabilities for the most probable and second most probable matches) was used to assess the uniqueness of the strongest match. Samples which fail the criteria 1/p < 100 and IF < 10 [13] are rejected. When both thresholds are exceeded, the proposed date is considered. This may involve taking into account the context of the sample and its relationship to other data, series length, timber quality and preservation, presence of juvenile or disturbed growth, geographic location relative to the reference chronology, and other dating evidence that may be available. A detailed discussion of ISODATE and the implemented statistical framework for δ^18^O dating can be found in [13,41].

## Results

### The TWR δ^18^O chronology

**Fig 2:**
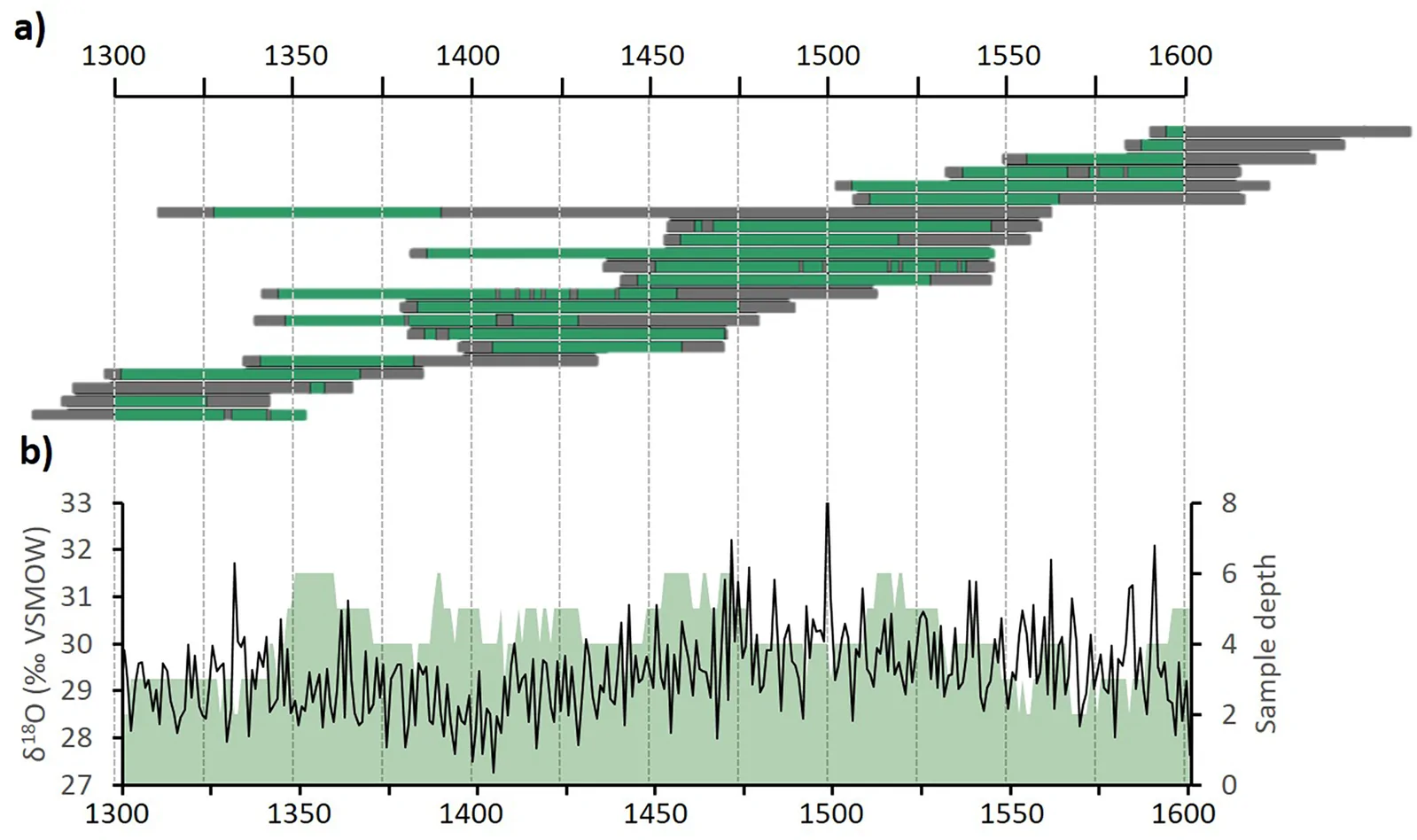
Development of the TWR chronology. a) Temporal coverage of the 22 samples used to construct the TWR δ^18^O chronology. Green bars indicate segments included in the final chronology; grey bars correspond to dated portions that were not analysed for stable isotopes (see Methods) b) The chronology represents the measured annual δ^18^O values (‰ VSMOW) of pooled latewood α-cellulose samples (black line). The green shaded area indicates the number of pooled samples by calendar year CE.

The TWR δ^18^O chronology covers a continuous period of 300 years, from 1300 to 1600 CE. Initially developed as a proof of concept for isotope dating in this region, this series currently comprises material from 22 trees (Fig 2a). The highest sample replication (4–6 trees) was achieved between 1349 and 1539 CE and therefore represents the most robust segment of the chronology (Fig 2b). This level of replication is generally considered sufficient to capture the common site-level isotope signal in homogeneous stands [44]. Outside this interval, replication decreases towards both ends of the series, where additional sampling is expected to further strengthen the common signal and improve dating capability.

### Shared signal across Europe

**Fig 3:**
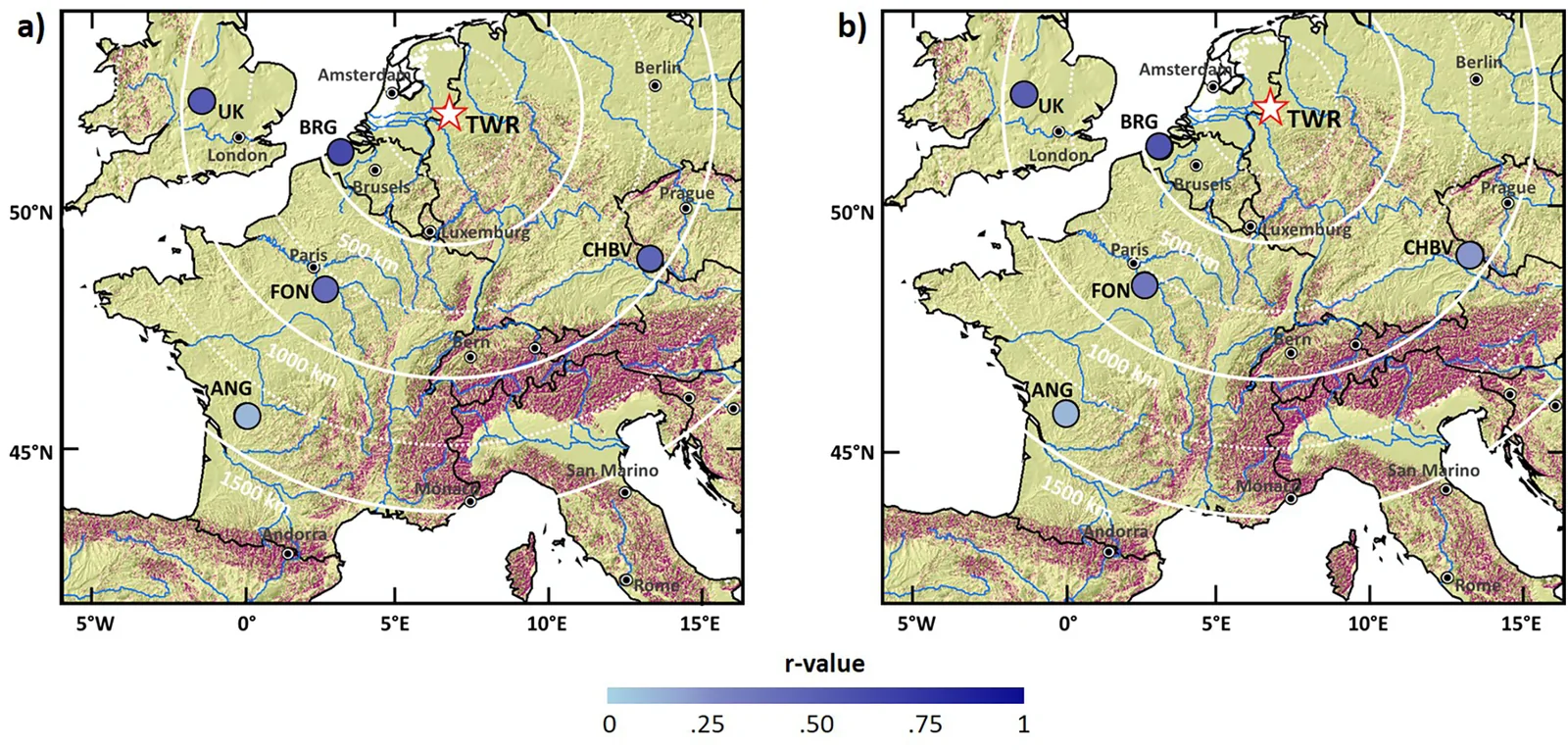
Spatial correlation between the TWR δ^18^O chronology and five independent tree-ring δ^18^O chronologies across Western and Central Europe. The star indicates the estimated wood source of the samples used in the TWR δ^18^O chronology. White concentric lines represent 250 km intervals from this point. a) Correlation for all the period available for each chronology (see Table 1 for information about time periods); b) Correlations for the common period shared by all the chronologies (1360–1468). Base map created by the authors using publicly available geospatial datasets. National boundaries from GISCO (Eurostat), river network from Natural Earth, and hillshade from the European Environment Agency (EEA). Thematic layers were generated by the authors.

We assessed the common signal between the TWR δ^18^O chronology and five European δ^18^O records (Fig 3), which differ in temporal coverage and sample replication, and explored the stability of this relationship through time. For the full overlapping period (correlation_1), the highest correlation (r = 0.59) is observed with the Bruges site chronology (BRG), followed by the central England chronology (UK) (r = 0.52) (Table 1; Fig 3a). Moderate correlations are found with chronologies from Fontainebleau (FON) (r = 0.45) and Czech-Bavaria (CHBV) (r = 0.43), while the lowest r-value is with Angoulême (ANG) (r = 0.11). For the common interval shared by all chronologies (correlation_2; 1360–1468 CE) (Table 1; Fig 3b), r-values are still highest for BRG (r = 0.56) and UK (r = 0.50), followed by FON (r = 0.36) and CHBV (r = 0.25).

**Table 1:** Correlation summary between the TWR δ^18^O chronology and the five independent tree-ring δ^18^O chronologies from Western and Central Europe for (i) a variable overlap and time range depending on data availability and (ii) the common overlap between 1360–1468 covering 109 years. Pearson’s correlation coefficient (r), degrees of freedom (df), and the p-values. Prior to correlation analysis, all chronologies were treated with a rectangular 9-year filter [14].

Variable overlap and range						Common overlap 109 years – Range 1360–1468		
	Overlap (years)	Range	r	df	p-value	r	df	p-value
BRG	137	1325–1468	0.59	119	**p < 0.001**	0.56	94	**p < 0.001**
UK	301	1300–1600	0.52	256	**p < 0.001**	0.50	81	**p < 0.001**
FON	275	1326–1600	0.45	235	**p < 0.001**	0.36	92	**p < 0.001**
CHBV	301	1300–1600	0.43	262	**p < 0.001**	0.21	76	0.03
ANG	241	1360–1600	0.11	92	0.063	0.1	36	0.17

**Fig 4:**
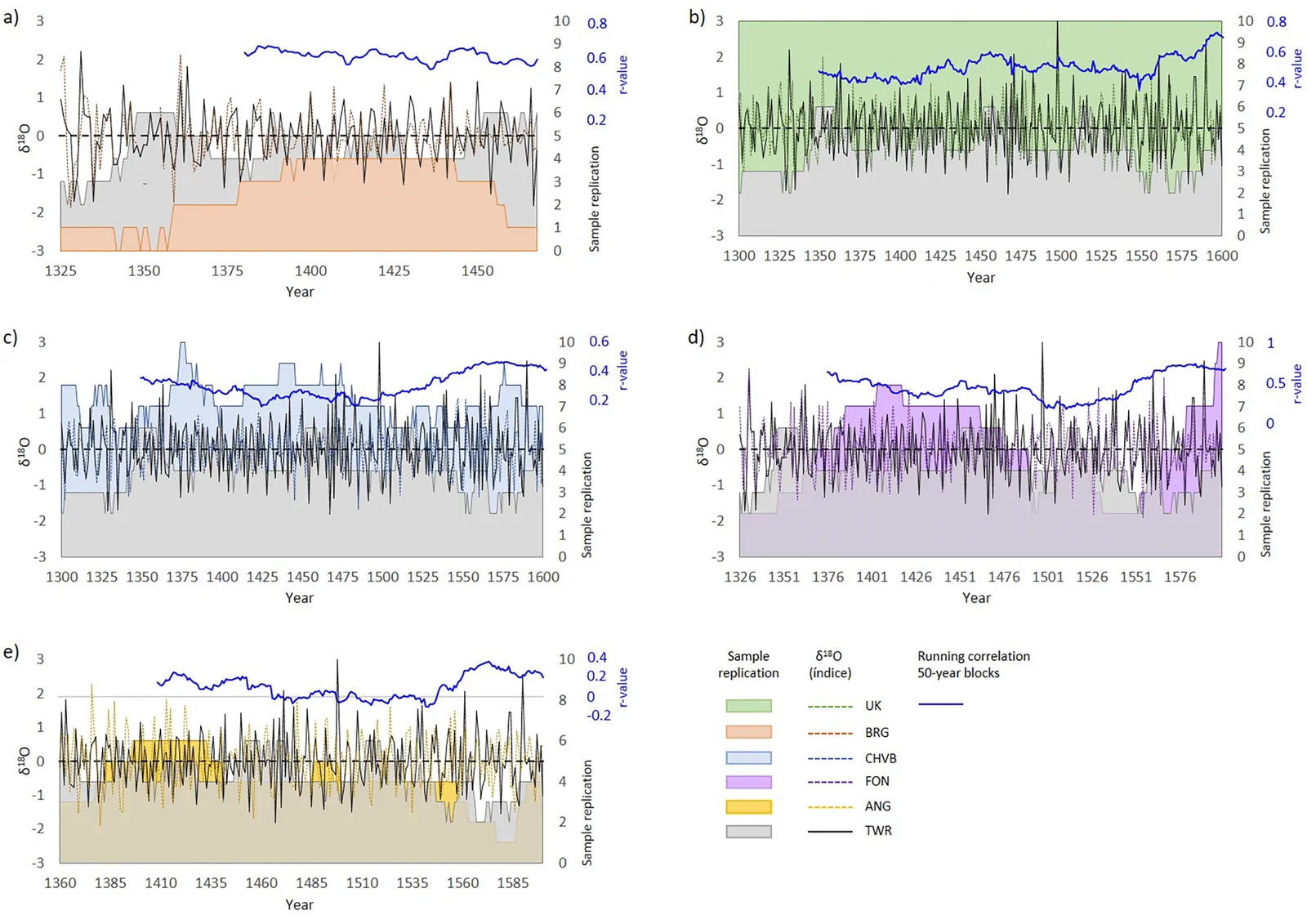
Running correlations (blue line in the top) calculated for 50-year between the Dutch oxygen isotope chronology (TWR; black line) and five European reference chronologies. a) BRG, b) UK, c) FON, d) CHBV, and e) ANG. For each panel (a–e), the two δ^18^O chronologies are shown as filtered annual values, together with their respective sample replication (coloured shaded areas, right y-axis).

To examine the influence of replication on the strength of the shared signal, we further analysed running correlations between TWR and the five reference chronologies (Fig 4). The UK chronology is the best replicated (N = 10 through the common period used in the analysis), whereas BRG is the one with the lowest replication. This analysis revealed that all correlations are statistically significant at p < 0.05 after accounting for autocorrelation, except for ANG, for which significance is only observed between 1564 and 1583. Correlations are predominantly positive, but their strength fluctuates through time and differs among regions. The correlations with a lesser degree of fluctuation through time occur with BRG (Fig 4a; r-values 0.51–0.65) and UK (Fig 4b; r-values 0.35–0.73). The correlation variability is higher for FON (Fig 4c; r-values 0.09–0.71), CHBV (Fig 4d; r-values 0.07–0.72), and for ANG (Fig 4e; r-values −0.1–0.36). Interestingly, an increase in correlation is observed for all chronologies (except for BRG) towards the third quarter of the 16th century, a period when the sample replication in the TWR chronology is lower.

### Testing the dating potential with samples from Jerome’s old church

**Fig 5:**
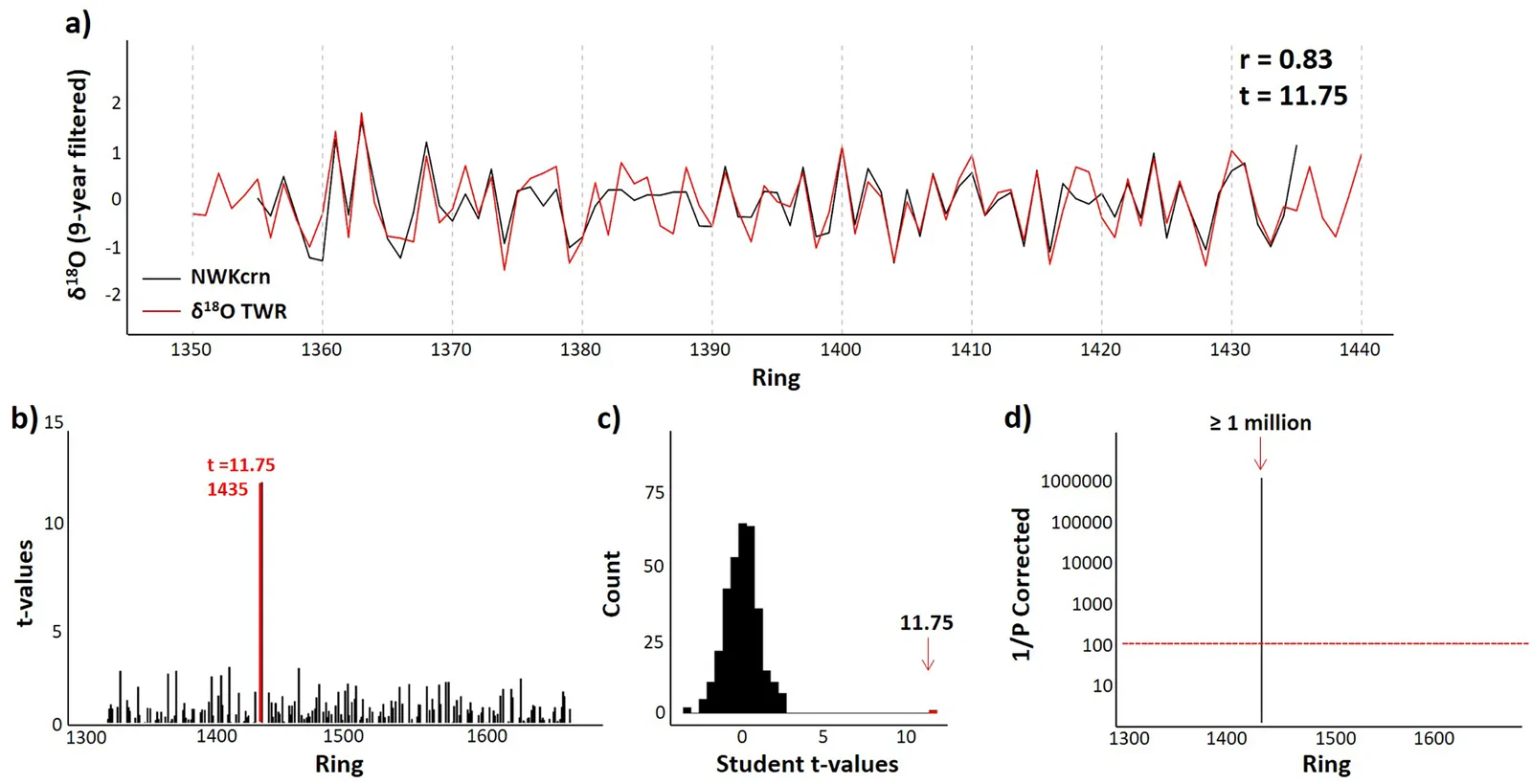
Jerome’s Old Church dating statistics and profile. Figures modified from ISODATE v2.0.2 [41]. a) Filtered TWR δ^18^O indices plotted along with the Noordwijk composite chronology (NWKcrn) with the corresponding Pearson’s correlation coefficient (r) and Student’s t. b) Distribution of Student’s *t*‐values through time with best match at the year 1435. c) Histogram showing the distribution of all annual matches derived from the dating procedure with the best match (1435) and Student’s t‐value identified. d) Corrected probabilities for the dated year satisfying the dating thresholds (1/p ≥ 100 and Isolation Factor ≥ 10 [13]). The dashed horizontal red line represents the Bonferroni‐corrected probability of at least one in one hundred (1/p ≥ 100).

The timbers from Jerome’s Old Church in Noordwijk represent an ideal case study to test the potential of the new TWR δ^18^O chronology for dating purposes. The comparison of the isotopic site-chronology developed from five oak timbers from Noordwijk’s church (δ^18^O NWKcrn [29]) with the TWR δ^18^O chronology in ISODATE v2.0.2 [41] revealed a single position where NWKcrn and TWR δ^18^O chronology match, providing an independent validation of the correct dating of the NWKcrn chronology, which covers the period 1355–1435 (Fig 5). Pearson’s correlation coefficient at this alignment is 0.83, along with a Student’s *t*-value of 11.75. The visual comparison of NWKcrn with TWR supports the statistical results (Fig 5a). This date shows the strongest statistical alignment across the entire testing range (Fig 5b, c) with a corrected 1/p value of ≥ 1 million for the match at 1435 CE (Fig 5d). Moreover, the next highest match fell far below this level, resulting in an isolation factor (IF) greater than 10. These measures exceed both criteria for rejection of a potential date: 1/p ≥ 100 and IF ≥ 10 [13], and indicates that the match at 1435 CE is highly unlikely to have occurred by chance. These results fully agree with the independent dating obtained through the isotopic series of the church, initially dated by conventional ring-width dendrochronology [29].

### Spatial effect on the dating of individual and coeval δ^18^O series

**Fig 6:**
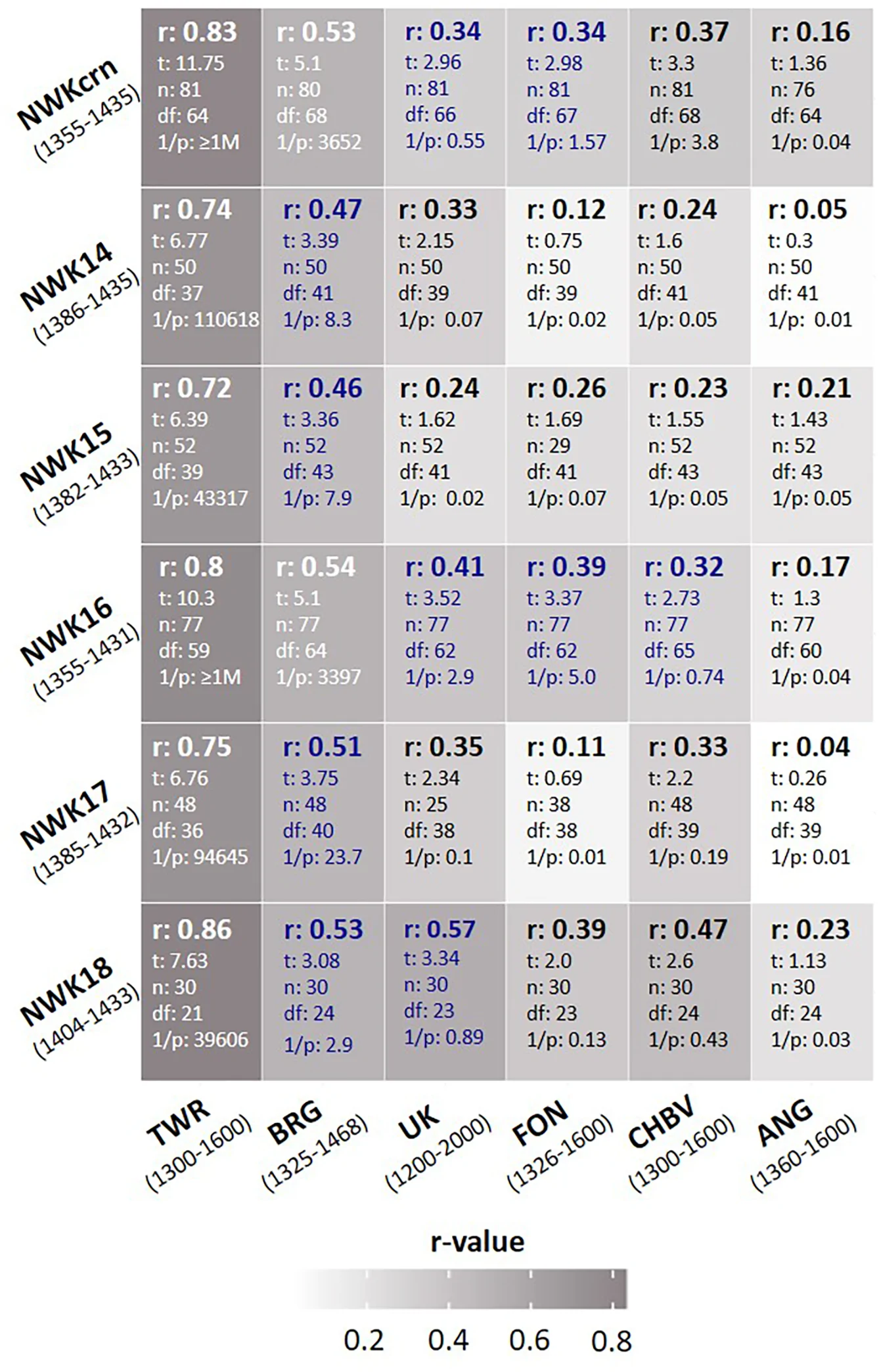
Correlation matrix (r-values) between the NWK composite chronology (NWKcrn) and individual NWK series positioned at the *a priori*-dated position, against the newly developed TWR chronology and other available European reference chronologies. For each comparison, the Pearson correlation coefficient (r), *t*-value (t), sample length (n), degrees of freedom (df), and probabilities (1/p) are shown. White text indicate that the *a priori*-dated position was the first match suggested by ISODATE and fulfilled the applied dating criteria [13,41]. Blue text indicates that the *a priori*-dated position was the first ISODATE match but did not meet all statistical thresholds required for confident dating. Black text indicates that the *a priori*-dated position not ranked as the first match by ISODATE. The grayscale shading reflects the strength of the correlation (r-value).

To understand the geographical spread of the isotopic signal for dating purposes in the Euro-Atlantic region, we tested whether the Noordwijk composite chronology (NWKcrn) and the isotopic series from the individual timbers could have been dated with other existing oxygen isotope chronologies from Northern or Central Europe (Fig 6).

The correlation analysis between the Noordwijk composite (NWKcrn) and TWR plus the other European isotope chronologies used in this study shows the highest match with TWR, as shown in Fig 5a, followed by BRG (r = 0.53). For the UK and FON chronologies (r = 0.34), ISODATE returned the actual dating position as the best match although the date did not pass the thresholds for consideration [13], and for CHBV (r = 0.37) and ANG (r = 0.16) the dating failed to meet the statistical thresholds for consideration (see full results in S1 File).

The dating exercise of all the individual δ^18^O series from Noordwijk with the chronologies returned their actual dating position as the best true match with the TWR chronology (Fig 6), supporting their temporal alignment. However, NWK16, the longest series with 77 rings passed the statistical tests for consideration of a data against one of the other chronologies (BRG, the geographically nearest; r = 0.54) (Fig 6). The rest of the individual NWK series failed to pass the tests in their correct dating position. For NWK16, the correct date was also proposed as the best match (while failing to meet thresholds) against UK, FON and CHBV, and for NWK18 (the shortest series with 30 rings) also with the UK (S1 File).

## Discussion

The main challenges for dendrochronological dating of historical timbers of oak throughout the Euro-Atlantic region relate to the wood material itself (often showing few and/or complacent tree rings [22–24]), and to the uncertain or unknown provenance of the material [6,9,11]. The majority of timbers in this geographical area remain undated due to the limitations of ring-width dendrochronology, which requires long tree-ring series for robust statistical crossdating, sensitive growth-patterns (with high year-to-year variation) and well-established reference chronologies from the source region [6,10,11].

In recent years, several studies from the Euro-Atlantic region have demonstrated the potential of stable oxygen isotope dendrochronology to overcome limitations of conventional ring-width dating. Archaeological samples from Scotland [45], Belgium [22], and the Basque Country [20] have been successfully dated against isotopic reference chronologies developed in Central England [13] and France [42], reflecting the broader spatial coherence of the isotopic signal. However, this supra-regional consistency is not universal [20], and it remains unclear under which conditions local reference chronologies become necessary.

Our study contributes to this discussion in two main ways. First, we present a new stable oxygen isotope reference chronology developed from historic (building) timbers in The Netherlands, originally sourced from the Twente–Westphalia region in the eastern Netherlands/northwest of Germany, and evaluate its shared signal with other European isotope chronologies. Second, using pre-dated timbers from Jerome’s Old Church, we assess the statistical strength of their isotopic match at the calendar position independently obtained by comparing the individual series with our newly developed chronology first, and subsequently with other European references. In doing so, we revisit the question of whether a local isotope chronology may provide a clearer or more robust signal, particularly in situations where only a limited number of timbers (or even a single sample with few rings) are available to date an archaeological site or a historic building.

From a regional perspective, the newly developed TWR δ^18^O chronology shares varying degrees of signal with other European δ^18^O tree-ring records across both space (Fig 3) and time (Table 1; Fig 4). Spatially, a clear radial gradient is observed: correlation coefficients decrease with increasing distance from the inferred source area of the TWR timber. This pattern with a coherent hydroclimatic signal across north western and central Europe, likely linked to large-scale atmospheric circulation [46], which translates into high dating potential through isotopes [18]. In contrast, the weaker correlation with Angoulême, in south western France, indicates that its isotopic variability is influenced by different climatic drivers [47].

Angoulême lies within a transition zone between northern Europe and the western Mediterranean, two of the sub-regions identified within the ISONET network [34], each characterised by distinct precipitation-related δ^18^O signatures in tree-ring cellulose. The divergence between northern and south western chronologies may reflect a dipole-like structure between Northern and Mediterranean Europe [48], associated with pressure anomalies centred over the North Sea and an opposing pattern over the northern Mediterranean [49]. This configuration, resembling the European summer blocking pattern [50,51] and often linked to the Summer North Atlantic Oscillation [52], shapes the spatial distribution of summer temperature and precipitation. Nevertheless, Angoulême and Fontainebleau remain generally well correlated over the study period, except for a marked divergence around 1550 [42].

When the temporal evolution of correlation coefficients is examined alongside changes in sample depth (Fig 4), the spatial gradient becomes less straightforward: correlations with TWR strengthen markedly for several chronologies (UK, FON, CHBV, ANG) during the third quarter of the sixteenth century, a pattern not systematically linked to changes in replication and therefore likely reflecting additional factors.

Although chronology replication is low during some parts of the record and therefore warrants cautious interpretation, this increase in inter-chronology correlations coincides with a period of documented hydroclimatic change in western and central Europe. One plausible explanation is that a shift towards drier spring–summer conditions in west-central Europe around 1570, described using European oak ring-width chronologies [53], may have enhanced the climatic control on δ^18^O variability and increased spatial coherence among geographically distant regions. Drier conditions can strengthen the climatic sensitivity of δ^18^O in tree rings, potentially explaining the observed homogeneity of isotopic signals during this interval [42]. This interpretation remains tentative, but is consistent with previous dendroclimatological studies.

Pooling material to construct initial reference chronologies represents a cost-effective strategy to establish a regional signal, as demonstrated in the development of UK isotope chronology [13]. However, while this approach maximises spatial footprint, it inevitably reduces local specificity, hindering the availability of individual series for further testing. When individually measured δ^18^O series are available, re-clustering into local isotopic groups may enhance fine-scale dating and dendroprovenancing potential. The current limited number and broad spatial extent of European isotope chronologies constrain a detailed assessment of provenance capacity. Successful cases, such as the confirmation of the Basque origin for the Newport Medieval Ship timbers after testing English, French, and Basque isotopic data [20,54], demonstrate the promise of this approach.

Regarding the dating of the timbers from Jerome’s Old Church, the TWR chronology proved effective in dating all Noordwijk series, whose lengths range between 30 and 81 rings. However, this test was conducted within a single interval (1355–1435), corresponding to one of the better-replicated sections of the chronology (sample depth between six and four). Further testing using independent material from additional sites and different time intervals would help to better assess its performance across sections with lower replication. In any case, while replication may have supported the robustness of the match with TWR, our broader results indicate that dating performance cannot be explained by this factor alone. For example, although the UK chronology is the most highly replicated, none of the individual series from Noordwijk fulfilled the ISODATE dating criteria (Fig 6). Similarly, the dating success of the Noordwijk composite against BRG (chronology with the lowest replication), also seems to indicate that whilst it is an important factor, the success of isotopic dating is unlikely to depend on replication alone. Proximity to the wood source, in the Euro-Atlantic region, is also a positive factor.

The length of the samples to be dated also plays a role, as seen in the results of the dating exercise (Fig 6). When the individual Noordwijk series are compared with the chronologies, their correct date is returned with the Bruges chronology, but for all series except one (NWK16, 77 rings) the match does not meet the ISODATE selection criteria to be considered. This highlights the statistical limitations imposed by individual short series, which would require a local reference chronology to be dated, as demonstrated with the TWR chronology.

Our findings therefore reinforce previous demonstrations that stable oxygen isotope dendrochronology can successfully date short-lived, fast-grown timbers when an appropriate reference chronology is available [13,18,19,55,56]. However, they also highlight that the dating of short series depends on a combination of factors, including sample replication in the chronology, the temporal period, spatial representativeness of the reference chronology (i.e., proximity to timber origin), and the length of the isotopic series under investigation. Furthermore, the temporal variability underscores the importance of developing well-replicated δ^18^O reference chronologies with dense spatial coverage, allowing both stronger regional signals and the possibility of identifying intervals of enhanced or reduced supra-regional coherence. Such networks are particularly critical in archaeological contexts, where only a limited number of timbers may be available (and often with few rings).

Oxygen isotope dendrochronology offers a promising approach for dating historical oak timbers that cannot be securely dated using conventional tree-ring dendrochronology. In Europe, these include roof timbers [18,24], timbers from archaeological sites [5], and critically, ship timbers, which are central to the study of maritime archaeology, naval architecture, and historical trade [26].

Dating shipwreck timbers originating from regions with scarce, long, and well-replicated reference chronologies, such as the Balkans, Italy, or the Iberian Peninsula has been particularly challenging in the past. For example, in their analysis of 23 shipwreck assemblages [26], only 15% of the oak samples could be successfully dated, mainly because many timbers contained too few rings to support statistically robust cross-dating, substantially reducing the likelihood of obtaining secure dates using conventional dendrochronological approaches [8]. However, further testing across different regions, periods, and replication levels is required to assess its broader applicability and robustness.

## Conclusions and outlook

Fast-grown short-lived oak trees (<80 years) make up to 70% of the timbers in historic buildings in the Euro-Atlantic region dating from the 14th to the 17th centuries, and 85% in shipwrecks from the 15th to the 18th century. Up until now, our inability to provide exact dates for those timbers with conventional dendrochronology that relies on tree-ring widths has hindered the study of such a valuable resource of historical and environmental information. This study provides further evidence that δ^18^O is a promising dating method for unlocking this vast archive, enhancing our understanding of timber use, trade, and environmental history. This represents a significant research opportunity not only in Europe, but also for regions with similar timber resources and dendroclimatic constraints, such as parts of North America, East Asia (Japan, Korea, China), New Zealand and the Mediterranean.

The success of this study calls for a coordinated effort to develop and expand the network of tree-ring δ^18^O reference chronologies throughout Europe and beyond, adopting a harmonized approach to dating and chronology development. Some examples of these collaborative efforts are already underway across the research community in Spain [54], Japan [57], Korea [16], China [17,21], New Zealand [58,59] and USA [60] where isotopic dendrochronology is being used to date ancient artefacts and structures. In addition, work is currently ongoing to further strengthen and extend the TWR oxygen isotope chronology presented in this study.

Although isotope analysis requires additional laboratory preparation compared with conventional ring-width measurements, pooling strategies substantially reduce the number of isotope measurements required for chronology development. The integration of isotopic and traditional ring-width chronologies into unified dating frameworks, such as the ISODATE application [41], offers a promising path forward for the field. This will require close collaboration between dendrochronologists, isotope geochemists, archaeologists, and data scientists to ensure the standardization, interoperability, and long-term accessibility of these valuable datasets.

## Supporting information

S1 File Complete ISODATE dating results for the Noordwijk individual timber δ^18^O series. Microsoft Excel file containing the complete output of the ISODATE analyses performed on each individual Noordwijk timber using the five European reference δ^18^O datasets (UK, BRG, FON, ANG, and CHBV). Separate worksheets provide the full dating results obtained with each reference chronology, including all statistically evaluated candidate dates.
(XLSX)
